# Sequence Variability of pXO1-Located Pathogenicity Genes of *Bacillus anthracis* Natural Strains of Different Geographic Origin

**DOI:** 10.3390/pathogens10121556

**Published:** 2021-11-29

**Authors:** Yulia Goncharova, Irina Bahtejeva, Galina Titareva, Tatiana Kravchenko, Anastasia Lev, Ivan Dyatlov, Vitalii Timofeev

**Affiliations:** 1 State Research Center for Applied Microbiology and Biotechnology (SRCAMB), Obolensk 142279, Russia; iulia.belay@yandex.ru (Y.G.); bahtejeva@mail.ru (I.B.); titarevag@mail.ru (G.T.); tbkrav@mail.ru (T.K.); ivan_a_diatlov@mail.ru (I.D.); 2 The Weizmann Institute of Science, Rehovot 76100, Israel; anastasia.lev@weizmann.ac.il

**Keywords:** *Bacillus anthracis*, pathogenicity factors, genotyping

## Abstract

The main pathogenic factor of *Bacillus anthracis* is a three-component toxin encoded by the *pagA*, *lef*, and *cya* genes, which are located on the pXO1 plasmid. The *atxA* gene, which encodes the primary regulator of pathogenicity factor expression, is located on the same plasmid. In this work, we evaluated the polymorphism of the *pagA*, *lef*, *cya*, and *atxA* genes for 85 *B. anthracis* strains from different evolutionary lineages and canSNP groups. We have found a strong correlation of 19 genotypes with the main evolutionary lineages, but the correlation with the canSNP group of the strain was not as strong. We have detected several genetic markers indicating the geographical origin of the strains, for example, their source from the steppe zone of the former USSR. We also found that strains of the B.Br.001/002 group caused an anthrax epidemic in Russia in 2016 and strains isolated during paleontological excavations in the Russian Arctic have the same genotype as the strains of the B.Br.CNEVA group circulating in Central Europe. This data could testify in favor of the genetic relationship of these two groups of strains and hypothesize the ways of distribution of their ancestral forms between Europe and the Arctic.

## 1. Introduction

Anthrax is a particularly dangerous infection caused by the spore-forming Gram-positive bacterium *Bacillus anthracis* [1]. The main feature of the systematic position of *B. anthracis* is its high genetic similarity with some other bacilli—*B. cereus*, *B. thuringiensis*, *B. mycoides*, *B. pseudomycoides*, *B. weihenstephanensis*, *B. cytotoxicus*, and *B. toyonensis*. These species form a group called *Bacillus cereus* sensu lato, or *Bacillus cereus* complex [2]. Their genetic similarity provides grounds for considering this entire group as one unique species, which consists of groups of strains that differ from each other by the presence of some genetic markers, mainly plasmids and regulatory genes [2,3,4]. For *B. anthracis*, these determinants are primarily two plasmids: pXO1 and pXO2. pXO2 harbors genes (*capBCAD* operon) that encode enzymes for the synthesis of a capsule consisting of poly-D-γ-glutamic acid. This capsule covers the surface of the *B. anthracis* vegetative cell and protects it from host immune responses [5]. pXO1 harbors the genes of the tripartite anthrax toxin complex components: protective antigen (PA, encoded by *pagA*), lethal factor (LF, encoded by *lef*), and edematous factor (EF encoded by *cya*). PA is a pore-forming protein that forms complexes with LF or EF, called lethal toxin (LT) and edematous toxin (ET), respectively, and allows the effector proteins LF or EF to enter the cytosol of mammalian cells. LF is a Zn-dependent metalloproteinase that is specific for mitogen-activated kinases (MAPKKs or MEKs). The LF-caused cleavage of MAPKKs disrupts the signaling pathways of the host cells. EF is a calmodulin-dependent adenylate cyclase, which also disrupts the signaling pathways of host cells by increasing the level of synthesis of cyclic adenosine monophosphate (cAMP). Clinically, the effect of EF is manifested as tissue edema, which served as the basis for the name of this protein [6,7,8]. The emergence of these pathogenicity factors required a change in the protein expression regulation system. In *B. anthracis*, this happened through two genetic events: first, the inactivation of the global transcriptional regulator PlcR via a point nonsense mutation, which regulates, among other things, the expression of the pathogenicity factors of other *B. cereus* complex species; and second, the acquisition of a new transcriptional regulator AtxA. The gene *atxA* is located on the pXO1 plasmid. AtxA, directly and indirectly, controls the expression of more than a hundred genes localized on the chromosome and on both pathogenicity plasmids, including genes for capsule and toxin synthesis [9,10]. AtxA is critically important for the anthrax microbe to realize its pathogenic potential. Like the toxin and capsule, AtxA could be called a pathogenicity factor.

pXO1 and pXO2 are traditionally considered specific for *B. anthracis*; nevertheless, some *B. cereus* strains that possess pXO-like plasmids have recently been discovered. Some strains were identified as a separate phylogenetic unit: *B. cereus* biovar anthracis [11,12,13]. *B. cereus* biovar anthracis and *B. cereus* possessing pXO-like plasmids still differ from the anthrax microbe primarily in their pathogenicity. Such strains seem to be less pathogenic for humans than *B. anthracis*, as only a few clinical cases have been described [13]. However, at the same time, they are virulent for other large animals, including great apes and laboratory animals [11,12,13].

Acquisition of the pathogenicity factors described above and formation of the *B. anthracis* species occurred, according to modern estimations, 12–26 thousand years ago [11,14], so the global population of the anthrax microbe did not have time to divide into subspecies or any other small groups that would differ phenotypically. Nevertheless, several molecular typing methods make it possible to distinguish genetically different groups of strains. One of the most widely used methods for dividing the *B. anthracis* species into phylogenetic groups is typing based on a small set of so-called “canonical” single nucleotide polymorphisms (canSNP). *B. anthracis* strains are divided among canSNP-groups into three main evolutionary lines: A, B, and C [15].

The A lineage is the most genetically diverse; it includes the canSNP groups A.Br.008/009, A.Br.001/002, A.Br.Ames, A.Br.003/004, A.Br.005/006, A.Br.Aust94, A.Br.Vollum, and A.Br.WNA. Strains of these groups were found worldwide, but some of their geographical distribution patterns can be distinguished [16,17]. The most widespread group is the canSNP A.Br.008/009 group, also referred to as Trans-Eurasian (TEA), the strains of which circulate in Eurasia and are very widely spread in Russia. This group is divided into two subgroups: A.Br.008/011 and A.Br.011/009 [18]. Group A.Br.001/002 is widespread in China (central and eastern regions) and is the ancestor of the small group A.Br.Ames, which was brought to the United States from China [19].

Groups A.Br.WNA and A.Br.003/004 are widely represented in North and South America; group A.Br.005/006 is found in Australia, Africa, and Europe; group A.Br.Aust94 is distributed in Southeast Asia, India, Australia, western China, and Turkey; and the A.Br.Vollum group is spread across South Africa and some regions of Asia (Pakistan and Afghanistan) [16]. The B lineage is more limited in its distribution area. It includes the canSNP group B.Br.CNEVA, found in Central Europe; B.Br.Kruger, located in South Africa; and B.Br.001/002, the strains of which are spread across South Africa, Europe, and Korea, and which were recently also found in the Russian Arctic [16,20]. The C lineage includes only the canSNP group C.Br.001, which is occasionally found in North America. It is believed that this lineage evolved the slowest of all; therefore, it is the most genetically close to the putative common ancestor of all *B. anthracis* lineages and the most distant from the rest of the anthrax microbe population [15,21].

The C lineage is distinguished by having the lowest evolutionary rate, the most significant degree of genetic difference from the rest of the populations of the anthrax microbe, and being the closest to the putative common ancestor of all *B. anthracis* lineages [15,21].

Such a scheme of *B. anthracis* global population genotyping is quite convenient. However, it is not entirely exhaustive. Therefore, additional SNPs are often proposed for a more detailed phylogenetic analysis, making it possible to isolate new phylogenetic and phylogeographic groups [22,23]. However, mutations in the genes encoding pathogenicity factors have practically not been used before as additional markers for genotyping. Moreover, unlike many other pathogens [24,25,26,27,28,29], the allelic polymorphism of these genes in the anthrax microbe is practically not described. Only a few articles have been published in which the allelic polymorphism of individual pathogenicity genes was assessed among a small number of strains [30].

In this work, we attempted to describe the allelic polymorphism of *pagA, lef, cya*, and *atxA* genes among the *B. anthracis* strains from our collection and to analyze the data obtained in terms of their correlation with strains belonging to a particular evolutionary lineage and canSNP group, as well as with the geographic origin of these strains.

## 2. Materials and Methods

### 2.1. Strains and Growth Condition

In this work, we used 40 live *B. anthracis* strains from the SRCAMB collection listed in Table 1 (State Research Center for Applied Microbiology and Biotechnology, Obolensk, Russia). All these strains were isolated in the territory of Russia and the former USSR. Vegetative cells of *B.anthracis* strains were cultured on BHI agar (SRCAMB), then inactivated, and sterile total genomic DNA was isolated using the Genomic DNA Purification Kit (Thermo Fisher Scientific, MA, USA). All manipulations were performed in a biosafety laboratory level 3. The sterility of the DNA samples was confirmed by cultivating 5% of the final DNA volume with negative results. The DNA concentration was quantified using the NanoDrop One^c^ spectrophotometer (Thermo Fisher Scientific).

Additionally, we used the whole-genome sequencing data deposited in GenBank for 48 strains of *B. anthracis* and *B. cereus*.

### 2.2. Whole-Genome Sequencing 

DNA libraries were prepared using the Nextera DNA Library Preparation Kit (Albiogen, Moscow, Russia). Whole-genome sequencing was performed using the Illumina MiSeq and Ion Torrent PGM instruments and the corresponding reagent kits: Ion PGM Reagents 400 Kit, Ion 318 Chip Kit (Life Technologies, Moscow, Russia), and MiSeq Reagent Kit v3 (Albiogen, Moscow, Russia).

### 2.3. Data Analysis

The assembling of the pXO1 plasmid sequence was performed using the DNASTAR software package (Lasergene, Madison, WI, USA) (https://www.dnastar.com/ perpetual license, accessed on 1 November 2021). The genome of the *B. anthracis* Ames Ancestor strain (plasmid pXO1, GenBank: AE017336.2) was used as a reference genome. The identified mutations and their coordinates were described in accordance with the reference genome. The in silico translation of nucleotide sequences into amino acid sequences was performed using the Transeq (EMBOSS, https://www.ebi.ac.uk/Tools/st/, accessed on 1 November 2021) The multiple alignments of nucleotide sequences were performed using the MEGA 7.0 software package (http://www.megasoftware.net, accessed on 1 November 2021). Simpson’s diversity index D was calculated using the PHYLOViZ 2.0 software [31]. Phylogenetic analysis was performed using the MEGA 7.0 (for UPGMA) and PHYLOViZ 2.0 (for goeBURST) software.

## 3. Results

Among the studied samples, we identified 11 alleles (sequence types, STs) of the gene *pagA* (D = 0.7717 (0.7231 ÷ 0.8202)), nine alleles each from the genes *lef* (D = 0.6468 (0.5585 ÷ 0.7352)) and *cya* (D = 0.6358 (0.5452 ÷ 0.7265)), and two alleles of the gene *atxA* (D = 0.023 (–0.0214 ÷ 0.0669)). All the STs differed from each other only by their single-nucleotide substitutions; we failed to find any deletions, insertions, or even substitutions of two nucleotides in a row. The identified STs, indicating their differences from the ST of the reference genome, are listed in Table 2, Table 3 and Table 4. Each gene ST to which the reference Ames Ancestor strain belonged was designated as ST1, then numbering was carried out in order of the decreasing number of strains of *B. anthracis*, after which STs, including strains of *B. cereus*, were numbered.

To assess the phenotypic manifestation of the identified nucleotide polymorphism—that is, whether the nucleotide substitution in each identified position is synonymous, leads to an amino acid substitution of the corresponding protein, or leads to its inactivation due to the appearance of a stop codon—we performed the in silico translation of nucleotide sequences into amino acid sequences. The coordinates of the predicted amino acid substitutions, indicating in which protein domain each substitution occurred, are shown in Table 5, Table 6 and Table 7. In these tables, the coordinates of amino acid substitutions are indicated for the complete sequence of translated proteins, excluding their post-translational modification, which consisted of the cleavage of the N-terminal signal sequence. In the PA protein, this is a 29 amino acid sequence; therefore, the mature protein length was reduced from 764 to 735 amino acids [32]. In LF and EF, this is a 33 amino acid sequence, so their length was reduced from 808 to 776 [33] and from 800 to 767 amino acids [34], respectively.

The tables do not show data for the *atxA*, since there is practically no polymorphism for this gene. Even the *B. cereus* strains 03BB102 and G9241, significantly different from *B. anthracis* strains, carry the same allele of *atxA* as the rest of the sample. The only mutation of this gene was found in the *B. cereus* biovar anthracis strain CI. The single-nucleotide mutation *atxA* 563T→A leads to the AtxA 188I→N amino acid substitution. Nevertheless, despite the extremely low degree of *atxA*, we did not exclude it from further work and used SNP *atxA* 563T→A as a marker, the presence of which emphasizes the genetic remoteness of the CI strain from the rest of the studied sample.

The division of the studied strains into genotypes (GT) based on a certain combination of studied genes STs is shown in Table 8. GTs were numbered according to the following principle; GT, which included the reference strain, was designated as GT1, and subsequent GTs were numbered in decreasing order of the number of strains in which they were detected, with the last numbers being assigned to the GTs of *B. cereus* strains. In total, we identified 19 genotypes (D = 0,8908 (0,8604 ÷ 0,9212)). More details on GTs are given in Table S1.

By describing the allelic polymorphism of pathogenicity genes localized on the pXO1 plasmid, identifying their sequence types, and dividing the strains of the studied sample by genotypes based on a certain set of sequence types of individual genes, we thus applied the MVLST method (Multi-Virulence Locus Sequence Typing).

In the present work, we limited our study to only the genes located on the pXO1 plasmid, not taking into account the genes of capsule synthesis; therefore, this molecular typing scheme can be designated as MVLST_pXO1_ genotyping, and individual genotypes can be designated as MVLST_pXO1_ GTs. It is noteworthy that the division of strains into the MVLST_pXO1_ GT, in general, repeats its division into canSNP groups. Even the diversity index is very similar—0,8908 (0,8604 ÷ 0,9212) for MVLST_pXO1_ and 0,8582 (0,8037 ÷ 0,9126) for canSNP typing. However, this observed pattern was not absolute; in some cases, strains of several canSNP groups formed a common GT, or, on the contrary, strains of one canSNP group were split into several GTs. Below, we provide a description of such exceptions.

GT1 includes 11 strains: 7 out of 12 strains of the canSNP group A.Br.001/002 and all four strains of the group A.Br.Ames. Five strains of the A.Br.001/002 group, not included in GT1, form GT7, which is distinguished by one nonsynonymous SNP *lef* 895G→T (LF299A→S).

GT2 includes 18 strains: 15 out of 32 strains of the line TEA (12 strains of the A.Br.008/011 canSNP group and three strains of the canSNP group A.Br.011/009), both strains of the A.Br.003/004 group, and one of the five strains of the group A.Br.005/006. All strains of the canSNP group A.Br.008/011 not included in GT2 (n = 17) form GT3, which differs from GT2 in two SNPs: *pagA* 981A→T and *lef* 2126A→G. Four strains of the A.Br.005/006 group, not included in GT2, form two separate genotypes: GT8 (n = 3), which has an additional unique non-synonymous SNP *cya* 2129A→C (EF 710H→P), and GT11 (n = 1), which lacks an SNP *cya* 600C→T specific for GT2.

GT5 includes seven strains: all five strains of the B.Br.CNEVA group isolated in Central Europe (except strain 44, whose place of isolation is unknown) and two strains of the B.Br.001/002 group isolated in the Russian Arctic. This genotype did not include the remaining five strains of the B.Br.001/002 group from the studied sample. Three of them were isolated in Siberia, the Baltic region, and Korea from GT9, which differs from GT5 by its additional unique nonsynonymous SNP *pagA* 1297A→G. Two other strains isolated in Sweden and South Africa form the separate GT10 due to the absence of SNP *pagA* 1799C→T. This SNP is also absent in the Kruger B strain, the only representative of the Br.Kruger group (GT15) in our sample. Instead of this SNP, GT15 has a unique SNP *pagA* 1765C→A.

Strains of the A.Br.Aust94 group also form two genotypes: seven of its eight strains were included in GT4, and the Ohio ACB strain is allocated into a separate genotype GT14 due to the presence of the unique synonymous SNP *pagA* 1803T→C.

**Figure 1:**
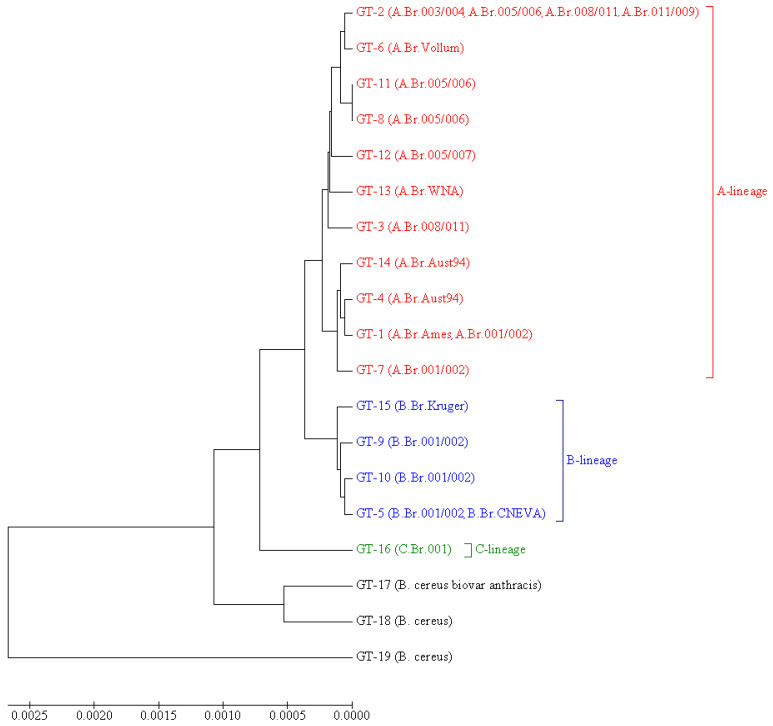
UPGMA dendrogram illustrating the phylogenetic relationships of MVLSTpXO1 genotypes. Opposite each GT, it is indicated in parentheses which canSNP groups this GT includes. For each GT, which includes strains that do not belong to *B. anthracis*, the species is indicated instead of the canSNP group. Braces and colors mark genotypes belonging to the different evolutionary lineages: A (red color), B (blue color), and C (green color).

The phylogenetic relationships of all the MVLSTpX1 genotypes described in this work are illustrated in Figure 1. As can be seen from this figure, and which is quite expected in view of the above, MVLSTpXO1 genotypes form clusters belonging to different evolutionary lines: A, B, and C. The most numerous «A» cluster is divided into two subclusters. The first of them combines GT1, GT4, GT7, and GT14—that is, strains of canSNP groups A.Br.001/002, A.Br.Ames, and A.Br.Aust94. The second one includes GT2, GT3, GT6, GT8, GT11, GT12, and GT13—i.e., strains of the canSNP groups A.Br.003/004, A.Br.005/006, A.Br.005/007, A.Br.Vollum, A.Br.WNA, and A.Br.008/009 (subgroups A.Br.008/011 and A.Br.011/009). GT16, which includes the only strain of C-lineage, is far from clusters A and B and, in fact, occupies an intermediate position between *B. anthracis* and *B. cereus* strains. *B. cereus* biovar anthracis is the most distant from the rest of the studied sample.

**Figure 2:**
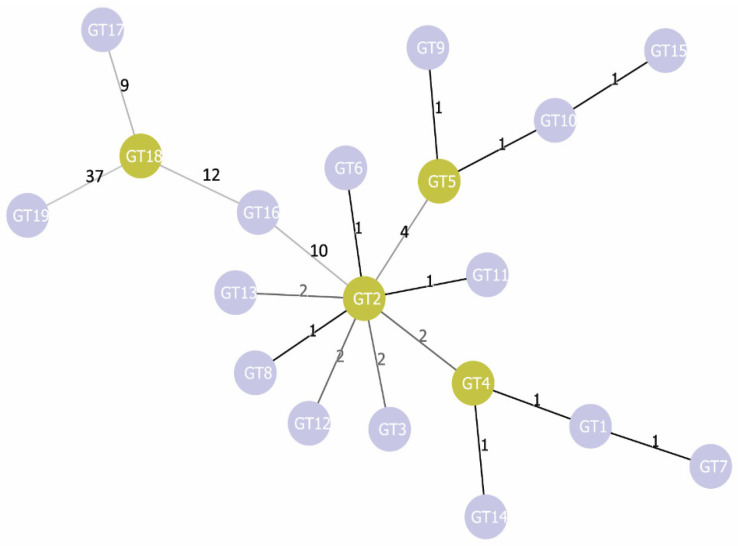
Phylogenetic tree of MVLSTpXO1 genotypes constructed using the goeBURST algorithm. The numbers above the line linking two genotypes indicate the genetic distance between these genotypes.

Additionally, we carried out phylogenetic analysis using the goeBURST algorithm [35] (Figure 2). As a result, we found that GT2 is linked simultaneously with nine genotypes: GT3, GT4, GT5, GT6, GT8, GT11, GT12, GT13, and GT16. This GT2 differs from GT1 by three SNP: *pagA* 195 C→T, *pagA* 1799 C→T, and *cya* 600C→T. All three SNPs are characteristic for most of the strains in our sample: synonymous SNP *pagA* 195C→T is found in 64 strains, SNP *pagA* 1799C→T (PA 600A→V) is found in 61 strains, and all of them also possess the SNP *pagA* 195C→T. The synonymous SNP *cya* 600C→T is found in 71 strains. All three SNPs together are found in 60 strains, but 42 of them have additional SNPs, due to which some other GTs are formed. Thus, we can assume that for the studied sample, GT2 with its three characteristic SNPs *pagA* 195 C→T, *pagA* 1799C→T, and *cya* 600C→T is the most typical; other GTs either lack these markers or acquire additional SNPs. In goeBURST analysis, all genotypes form three main clonal complexes (CC) unite genotypes, the genetic distance between which is minimal and equal to one (Figure 2). Two of them include some GTs of A lineage. The first one remains identical to the corresponding subcluster on the dendrogram shown in Figure 1 and includes GT1, GT4, GT7, and GT14—that is, canSNP groups A.Br.001/002, A.Br.Ames, and A.Br.Aust94. The second CC is formed by GT2, GT6, GT8, and GT11—that is, by the groups A.Br.003/004, A.Br.005/006, and A.Br.Vollum and the strains of the TEA line (groups A.Br. 008/011 and A.Br.011 / 009) lacking the SNP *pagA* 981A→T. The third CC is the most genetically distant from the first two and unites all genotypes of the B lineage (groups B.Br.001/002, B.Br.CNEVA, and B.Br.Kruger).

## 4. Discussion

As is known, anthrax mainly affects ungulates [1], including horses and livestock—that is, animals that are still the basis of agriculture, and until modern times were also the only land transport. As a result, *B. anthracis* could easily be introduced into different regions not only by the natural migrations of ungulates but also through human activities, such as colonization, military operations, and trade expeditions. In the course of such activities, not only did people themselves move, but so too did triding and sled animals; pack animals; livestock; and livestock products, such as leather, fur, woolen clothing, and harnesses, as well as items of ammunition. These animals could be sick with anthrax, and goods could be contaminated with *B. anthracis* spores, which are easily transported and remain viable. Perhaps the anthropogenic transportation of spores was the main driver of the spread of *B. anthracis* across the planet [14]. Due to the natural and anthropogenic transfer, genetically related strains may be introduced into distant regions, where their further evolution may proceed differently. At the same time, genetically distinct strains from different origins can be brought into the same area over and over again. As a result, local populations of the anthrax microbe can be represented by strains of different genetic lines. Therefore, the geographical origin of a particular *B. anthracis* strain is not very informative in itself, but rather should be considered in conjunction with its genetic profile, as well as the natural, historical, and economic characteristics of the region where this strain was isolated. Based on this, we attempted to identify any correlations between the geographical origin of the strains from our sample, their canSNP groups, and their MVLSTpXO1 genotypes.

In this analysis, the first thing that caught our attention was the synonymous SNP *pagA* 981A→T. This SNP had earlier been proposed as a specific marker for a small subgroup of the canSNP group A.Br.008/009, which was originally isolated in Russia (the so-called Sverdlovsk group) but has also been found in Norway, Hungary, and Slovakia [36]. A little later, Eremenko, during the genotyping of *B. anthracis* strains from the Stavropol Antiplague Institute collection, found this SNP in all eight strains belonging to the canSNP group A.Br.008/009 in the studied sample [37]. In the present study, we found SNP *pagA* 981A→T in 17 out of the 27 strains in the A.Br.008/011 group (as we have already indicated, A.Br.008/011 together with group A.Br.011/009 is included in the A.Br.008/009 group) isolated in the territory of the former USSR (Table 1, Table 8 and Table S1). Still, we did not find it in any strain from the comparison group—that is, isolated in the other regions. All 17 strains belong to MVLST_pXO1_ GT-3 (Table 8 and Table S1)—i.e., they have no other differences among themselves in terms of the sequence of genes described in this work. Thus, our results complement the data of Eremenko and Okinaka [36,37] and indicate that SNP *pagA* 981A→T is a marker of a rather large subgroup of the A.Br.008/011 group. This subgroup is spread over territory stretching over 6000 km from west to east and about 4000 km from north to south, including Central Asia, the Caucasus, the Black Sea and Caspian steppes, the European part of Russia, and even the eastern Arctic (Yakutia). Moreover, the A.Br.008/011 *pagA* 981A→T subgroup (or MVLST_pXO1_ GT-3) includes, if not the majority, then at least half of the strains of the A.Br.008/011 group circulating in the territory of the former USSR and the former Russian Empire. Additionally, from a deeper historical perspective, the GT3 strain distribution area coincides with the territories that, in the XII–XVIII centuries, were part of the Mongol Empire and the states into which it disintegrated (Golden Horde, Chagatai Horde, Ilkhanat), as well as the zones of their political influence (Novgorod region of Russia) and territories subjected to Mongol military campaigns (Hungary and Slovakia). Earlier, we hypothesized about the significant role of the Mongol invasion of the west in the spread of anthrax across Eurasia [20]. The geographical area of distribution of the subgroup A.Br.008/011 *pagA* 981A→T described in this article additionally supports our hypothesis. Even the fact that one of the strains of this subgroup was isolated in Norway fits well into the framework of this concept. The northeast region of Russia—Novgorod and Pskov principalities—through most of its history was associated with Scandinavian states not only by trade ties but also by a non-stop series of military conflicts, which may have led to the introduction of A.Br.008/011 *pagA* 981A→T strains into Scandinavia through the exchange of goods and/or war booty.

As mentioned above, in addition to *pagA* 981A→T we found several more SNPs dividing the strains of one canSNP group into different GTs. Additionally, there is some reason to link these GTs to a specific geographic region.

For example, the unique SNP *pagA* 1803T→C distinguishes strain Ohio ACB from other strains of the canSNP group A.Br.Aust94 in our sample. Additionally, this is the only strain isolated in the USA, while other strains of the group A.Br.Aust94 were isolated in the Old World. This data prompted us to evaluate the possibility of using SNP *pagA* 1803T→C to determine the origin of the strain of the A.Br.Aust94 group from North America. Not having such strains in our collection, we had to limit ourselves to a small number of deposited archives with the raw WGS data of the A.Br.Aust94 group isolated in the USA (Table 9). However, contrary to our hopes, we found this SNP in only two American strains out of ten. Therefore, the maximum that can be said about the diagnostic role of this SNP is that it occurs in some A.Br.Aust94 strains circulating in the United States.

Another exciting marker is SNP *lef* 895G→T, due to the presence of which five of the twelve strains of canSNP group A.Br.001/002 form a separate genotype GT7, while most of the strains of this group are included in GT1. We were interested in the fact that the strains with this SNP were isolated mainly in the New World—the USA, Brazil, and Jamaica—with a single one being isolated in Bangladesh. Strains lacking this SNP were isolated in Russia, China, Kazakhstan, and Germany. To find out whether the SNP lef895G→T is characteristic of strains of the A. Br.001/002 group of specifically American origin, we analyzed 42 additional genomes of *B. anthracis* strains of this group, of which three strains were of American origin, 28 were of European origin, and 11 strains were of Asian origin. We found this SNP in all three American strains, but also in 23 of 28 European and 8 of 11 Asian strains (Table 10).

Thus, SNP *lef* 895G→T was found to be typical for most strains of the A.Br.001/002 group, regardless of their geographic origin. Additionally, we can make some inferences about the absence of this SNP as a marker of a subgroup of the A.Br.001/002 group. This subgroup has the same GT as the A.Br.Ames group, therefore, is more related to it (within the framework of the typing scheme used). Considering that the A.Br.Ames group is descended from the A.Br.001/002 group [19], it can be assumed that the GT1 subgroup of the A.Br.001/002 group is a kind of “transitional link” that has already acquired plasmid markers of the A.Br.Ames group, which has not yet developed the chromosomal canSNPs of this group.

Unfortunately, unlike the A.Br.008/011 *pagA* 981A→T subgroup, we do not have data that could somehow explain the reasons behind and patterns of distribution of the A.Br.Aust94 *pagA* 1803T→C and A.Br.001/002 *lef* 895G→T subgroups.

Interestingly, *lef* 895 is the only position that we found that possesses three allelic states—G, T, and A. *lef* 895G is found in most strains; *lef* 895A is a marker found in all canSNP strains of lineage B canSNP groups; and *lef* 895T, as indicated above, is a specific marker of the GT7 subgroup of A.Br.001/002 strains. However, besides the group of A.Br.001/002 strains, *lef* 895T is unexpectedly found in a non-anthracis strain, *B. cereus* G924.

Moreover, we would like to focus on lineage B strains. Previously it was shown that *B. anthracis* strains of the B.Br.001/002 group circulating in Siberia form a separate phylogenetic cluster, named «Siberia» [38]. However, in our work two Siberian B.Br.001/002 strains were combined into one GT5 with the B.Br.CNEVA group, but not with other B.Br.001/002 strains, including not with the strain I-364 isolated in Siberia. Both of these strains were isolated in the Arctic. Strain Yamal-2 was isolated during an anthrax outbreak in Yamal in 2016, which occurred due to the thawing of soil anthrax conserved in permafrost. Strain LP53/5YA was found in permafrost during paleontological excavations in Yakutia. The very history of these strains, such as their isolation in the Arctic region, including isolation from the permafrost, indirectly indicates that their age is most likely older than that of other strains of this line. In this case, GT5 unites the B.Br.CNEVA group and the archaic strains of the B.Br.001/002 group and this GT was formed before the evolutionary divergence of these two canSNP groups. Moreover, the fact of the certain phylogenetic closeness of strains circulating in Central Europe with strains isolated in the Arctic, which is remote from Europe and is not connected to it by animal migration routes or economic ties, remains intriguing.

The similarity of strains from the Arctic and Central Europe raises questions not only about the past of these strains (how exactly and when they spread between these regions and within them) but also about their future. Global climate changes with a certain probability can lead to the melting of permafrost, and thawing of soil foci of anthrax, as it happened in 2016 in Yamal. Moreover, the random nature of the finding of Yakut strains illustrates that viable spores of *Bacillus anthracis* can be distributed in permafrost much more widely than it seems at the moment. A change of permafrost boundaries means not only the de-preservation of the microorganisms presented in it but also means an improvement of the conditions for the plants’ vegetation. In turn, this means an increase in the food supply of animals, which can potentially lead to a rise in the number of endemic animals and migrations of animals to the region from low-latitude areas. That would also create favorable conditions for increasing the livestock of farm animals, primarily deer, which are traditionally bred in a nomadic way, that is, the maximum number which directly depends on the productivity of pastures. Such an increase in the number of animals is likely to outpace the development of the region’s infrastructure, including medical and veterinary supervision, which could provide timely prevention of anthrax outbreaks. In epidemiological investigations of such episodes, any similarity of the Arctic strains with the Central European strains can play a cruel joke and lead to the mistaken impression that the strain that caused the outbreak is not endemic but brought from Europe.

In addition to describing the distribution of certain markers in phylogenetic and phylogeographic groups of *B. anthracis*, it would be interesting to try to find relationships between the MVLSTpXO1 genotype of such groups and its phenotypic manifestation. Since the function of the genes used in this genotyping scheme is the synthesis of toxins that directly affect the host organism, such a phenotypic manifestation could be differences in the pathogenicity of strains with different MVLST_pXO1_ genotypes. This approach is more or less applicable to other pathogens [24,25,26], but it is very difficult for the anthrax microbe.

It is hardly possible to identify differences in pathogenicity in natural conditions during anthrax outbreaks—due to modern sanitary and veterinary control, such outbreaks are eliminated by antibiotic therapy, the vaccination of at-risk groups, and the slaughter of sick animals before statistically reliable data can be collected. Therefore, any assumptions that we can make about the potential influence of the MVLSTpXO1 genotype are highly speculative and do not have proper experimental confirmation. Nevertheless, we consider it possible to state a number of such suggestions.

The first thing that can be noted is the presence of four vaccine strains in the studied sample—A16R, obtained in the 1950s in China [39]; Sterne, obtained in South Africa in the 1930s, which is widely used as a veterinary vaccine throughout the world [40]; V770-NP-1R, which has been used as a producer for the AVA vaccine in the United States since the 1970s; and STI-1, which has been used as a live vaccine for humans since the 1940s in the USSR and now in Russia. Thus, the sample includes the most widespread vaccine strains, obtained independently in different parts of the world. All four of these strains retained the pXO1 plasmid but lost the pXO2 plasmid. Our analysis shows that none of these strains acquired any peculiarities in the sequence of toxin synthesis genes that distinguish them from natural, completely virulent strains. Moreover, the A16R strain does not differ in terms of the sequence of these genes from its parent strain A16. Thus, if the attenuation of *B. anthracis* strains is caused by any changes in the genome, in addition to the loss of the capsule formation plasmid [41], then these changes do not affect the genes involved in toxin synthesis.

The second point regarding the comparative virulence of *B. anthracis* strains that we would like to discuss concerns the modeling of anthrax. Although, as we indicated above, it is hardly possible to reveal such facts using laboratory models, we found in the literature a description of one exception to this pattern. When guinea pigs were vaccinated with the AVA vaccine, it was found that during subsequent infection different strains of *B. anthracis* overcome post-vaccination immunity with different efficacies—the survival rate of animals varied from 6 to 100% [42]. The authors could not find a correlation of this feature of the strains with any other characteristics, including their geographical origin. However, here it is worth paying attention to the composition of AVA; it is a precipitate of the culture fluid of the nonencapsulated strain V770-NP1-R (belongs to A.Br.003/004 and GT2)—that is, the PA, LF, and EF of this strain act as immunogenic determinants [43]. If the LF, EF, and PA sequences of the infecting strain and the V770-NP1-R strain are different, then the antibodies generated after vaccination may poorly recognize the toxins of the infecting strain, which may lead to an increase in the death rate of the infected animals. In this case, the ability of a strain to overcome post-vaccination immunity may depend more on its toxin synthesis gene sequence—that is, the MVLSTpXO1 genotype—than on other characteristics, including the region where the strain was isolated. Unfortunately, we could not verify this assumption, since we could not find in the available sources any data that could be used to determine the MVLSTpXO1 of the strains used in [42]—the WGS data or at least their canSNP group, which, as we have shown in the present article, correlates with MVLSTpXO1 genotype.

However, whatever the interesting data obtained in the laboratory experiments are, they cannot fully reflect the natural virulence of the *B. anthracis* strains. For evaluating the hypothesis concerning the influence of the MVLST_pXO1_ genotype on the strain pathogenicity, the evidence collected during anthrax outbreaks among naturally susceptible animals (ungulates) is more important. We managed to find the results of an epidemiological analysis in the literature, which could be interpreted as indirect evidence of our hypothesis. When comparing two anthrax-endemic South African ecosystems—Etosha National Parks (Namibia) and Kruger National Parks (Republic of South Africa), which are home to large populations of zebras and Kudu—it was noted that in Etosha Park almost half of all reported cases of anthrax occur in zebras, while the share occurring in kudu is less than 1%. In Kruger Park, a diametrically opposite picture was observed—more than half of the cases were recorded in kudu, while zebras get sick far more rarely [1,44,45]. These two regions differ in terms of the biodiversity of the *B. anthracis* strains circulating there. In Etosha Park, strains of the A.Br.Aust94 group prevails, while strains of the A.Br.008/009 group are rarely found. Outside the park, in the territory of Namibia, strains of groups A.Br.005/006 and A.Br.001/002 have also been identified, while strains of B lineage have not been found in this country [46]. In the Republic of South Africa, the population of the anthrax microbe is more diverse; there are circulating *B. anthracis* strains of both A and B lineages belonging to the canSNP groups A.Br.003/004, A.Br.005/006, A.Br.Aust94, B.Br.Kruger, and B.Br.001/002 [47], while strains of B lineage (B.Br.Kruger and B.Br.001 002) were isolated directly in Kruger Park. This biodiversity is even used as an argument in favor of the South African origin of the *B. anthracis* species, although, in our opinion, it is just as likely to be due to the political and economic history of this region. South Africa has experienced a number of events, each of which could potentially be associated with the introduction of different strains of *B. anthracis* into the region through livestock and animal products. These include the migration of the Bantu pastoralist tribes from the north; two waves of European colonization from the Dutch (Boers) and the British; and two Anglo-Boer wars, in which riding and pack animals, supplies, and equipment for the warring parties were supplied from different countries. In addition, South Africa, due to its geographical position has been a major transit point for all European trade with the Indian Ocean region from the XVII century until the opening of navigation through the Suez Canal. In addition, it was South Africa, due to its geographical position, that was the main transit point for all European trade with the Indian Ocean basin from the 17th century until the opening of navigation along the Suez Canal. Such active movements of large masses of people, livestock, and goods, including livestock products, over several centuries, could lead to the contrasting high genetic diversity of *B. anthracis* strains in this region. Strains of the B lineage are currently circulating in South Africa. It could be assumed that these strains are capable of infecting different ungulates that differ evolutionarily and physiologically with greater efficiency than strains of line A, whether they are artiodactyl ruminants bovids (kudu) or non-artiodactyl monogastric equines (zebras). As one of the reasons for such a potential difference in virulence, we could suggest differences in the sequence of anthrax toxins in strains A and B, as shown in this article.

We are aware that our assumptions are too speculative and can hardly be tested in practice. If there is a difference in the manifestation of pathogenic properties in strains with the MVLST_pXO1_ genotype, then it is unlikely that it has a tangible epidemiological significance. Nevertheless, MVLST-typing, which primarily reflects the evolution of the main effector proteins, the acquisition of which turned *B. anthracis* into a dangerous pathogen, is of interest because at least in some cases it makes it possible to distinguish geographically separate groups of strains within the canSNP groups. The use of this typing method, especially in combination with other more common genotyping schemes, can to some extent bring us closer to a more complete understanding of the evolutionary patterns of the anthrax microbe and the distribution of its individual intraspecific groups across the globe.

## Figures and Tables

**Table 1:** Strains used in this work.

Strains from the SRCAMB Collection			Other Strains			
Strain *	Geographic origin	canSNP group	Strain *	Geographic origin	canSNP group	WGS data access number in GenBank **
I-271	Russia: Yamal Peninsula	A.Br.001/002	14RA5914	Germany	A.Br.001/002	CP023002.1
34(738)	Kazakhstan	A.Br.001/002	A16	China	A.Br.001/002	CP001971.2
52/33	Russia: Chechen Republic	A.Br.001/002	A16R	China	A.Br.001/002	CP001975.2
STI-1	Laboratory strain	A.Br.008/011	BFV	Jamaica	A.Br.001/002	CP007703.1
1273	Russia: Volgograd region	A.Br.008/011	FDAARGOS341	USA	A.Br.001/002	CP022045.2
53169	Missing	A.Br.008/011	SPV84215	Brazil	A.Br.001/002	CP019589.1
8(2099)	Russia: Tatarstan	A.Br.008/011	Stendal	Germany	A.Br.001/002	CP014177.1
LP51/4YA	Russia: Yakutia	A.Br.008/011	Sterne	USA	A.Br.001/002	CP009540.1
644/268	Ukraine	A.Br.008/011	Tangail-1	Bangladesh	A.Br.001/002	CP015777.1
1055/38	Russia: Samara region	A.Br.008/011	BA1015	USA	A.Br.003/004	CP009543.1
592/10	Moldova	A.Br.008/011	V770-NP-1R	USA	A.Br.003/004	CP009597.1
1183	Russia: Kabardino-Balkar Republic	A.Br.008/011	A0135	Tanzania	A.Br.005/006	SRR2968157
1298	Russia: Volgograd region	A.Br.008/011	CZC5	Zambia	A.Br.005/006	AP018444.1
1030/213	Russia: Karachay-Cherkessia	A.Br.008/011	K3	South Africa	A.Br.005/006	CP009330.1
1056/51	Russia: Stavropol territory	A.Br.008/011	A2075	Tanzania	A.Br.005/007	SRR2968187
219/6	Uzbekistan	A.Br.008/011	H9401	Korea	A.Br.005/007	CP002092.1
367/17	Russia: Tula region	A.Br.008/011	A2079	Tanzania	A.Br.005/008	SRR2968188
46/27	Russia: Chechen Republic	A.Br.008/011	Larissa	Greece	A.Br.008/011	CP012520.1
47/28	Russia: Chechen Republic	A.Br.008/011	PAK-1	Pakistan	A.Br.008/011	CP009324.1
48/29	Russia: Chechen Republic	A.Br.008/011	Turkey32	Turkey	A.Br.008/011	CP009316.1
531/17	Russia: Kalmyk Republic	A.Br.008/011	A1144	Argentina	A.Br.011/009	CP010853.1
546/714	Russia: Voronezh region	A.Br.008/011	London499	UK	A.Br.011/009	CP029806.1
68/12	Azerbaijan	A.Br.008/011	Pollino	Italy	A.Br.011/009	CP010814.1
7(992)	Russia: Novgorod region	A.Br.008/011	A0248	USA	A.Br.Ames	CP001599.1
914/213	Russia: Chechen Republic	A.Br.008/011	A2012	USA	A.Br.Ames	AE011190.1
LP50/3YA	Russia: Yakutia	A.Br.008/011	Ames Ancestor	USA	A.Br.Ames	AE017336.2
1(14) Stavropol	Ukraine	A.Br.008/011	Shikan-NIID	Japan	A.Br.Ames	AP014834.1
555/288	Russia: Orenburg region	A.Br.008/011	A3716	Namibia	A.Br.Aust94	SRR2968149
1199	Russia: Dagestan	A.Br.Aust94	K1285	Namibia	A.Br.Aust94	SRR2071843
1173	Russia: Stavropol territory	A.Br.Aust94	Kanchipuram	India	A.Br.Aust94	CP060195.1
1259	Russia: Stavropol territory	A.Br.Aust94	OhioACB	USA	A.Br.Aust94	CP009340.1
331/214	Azerbaijan	A.Br.Aust94	CDC684	USA	A.Br.Vollum	CP001216.1
822/7	Russia: Chechen Republic	A.Br.Aust94	SK-102	USA	A.Br.Vollum	CP009463.1
11(1940)	Turkmenistan	A.Br.Vollum	Vollum	USA	A.Br.Vollum	CP007665.1
15(1345)	Tajikistan	A.Br.Vollum	Vollum1B	USA	A.Br.Vollum	CP009327.1
LP53/5YA	Russia: Yakutia	B.Br.001/002	Canadian bison	Canada	A.Br.WNA	CP010321.1
Yamal-2	Russia: Yamal Peninsula	B.Br.001/002	BA1035	South Africa	B.Br.001/002	CP009699.1
I-364	Russia: Siberia	B.Br.001/002	HYU01	Korea	B.Br.001/002	CP008847.1
157(B-1107)	Estonia	B.Br.001/002	SVA11	Sweden	B.Br.001/002	CP006743.1
44	Data is missing	B.Br.CNEVA	17OD930	Switzerland	B.Br.CNEVA	CP029324.1
			BF1	Germany	B.Br.CNEVA	CP047132.1
			RA3	France	B.Br.CNEVA	CP009696.1
			Tyrol4675	Austria	B.Br.CNEVA	CP018904.1
			Kruger B	South Africa	B.Br.Kruger	GCA_000167295.1
			2002013094	USA	C.Br.001	CP009901.1
			*Bacillus cereus* 03BB102	USA	-	CP001406.1
			*Bacillus cereus* biovar anthracis CI	Ivory Coast	-	CP001747.1
			*Bacillus cereus* G9241	USA	-	CP009592.1

* strains belong to *B. anthracis* unless otherwise specified. ** access number to the sequence of plasmid pXO1 or similar (for *B. cereus* strains) or to the archive with raw WGS data.

**Table 2:** Sequence types of the gene *pagA*.

Sequence Type	Description of the Mutation	Number of Mutations in ST	List of Strains	Number of Strains	The Frequency of ST
ST1	-	0	14RA5914, A16, Tangail-1, Stendal, Shikan-NIID, Ames Ancestor, BFV, A0248, A2012, I-271, 34(738), 1259, 1199, 52/33, 331/214, 1173, 822/7, FDAARGOS 341, SPV842_15, A16R, Sterne, Kanchipuram, A3716	23	0.261
ST2	195 C→T, 1799 C→T	2	Tyrol 4675, Larissa, A1144, Canadian bison, BA1015, RA3, K3, Turkey32, Pollino, H9401, London 499, CZC5, 17OD930, BF1, LP51/4YA, LP53/5YA, 53169, 1273, 44, 8(2099), V770-NP-1R, PAK-1, 644/268, 1055/38, STI-1, 592/10, Yamal 2, K1285, A0135, A2075, A2079	31	0.352
ST3	195 C→T, 981 A→T, 1799 C→T	3	1(14)Stavropol, LP50/3YA, 68/12, 367/17, 531/17, 7(992), 1056/51, 46/27, 1030/213, 1183, 219/6, 47/28, 48/29, 914/213, 1298, 546/714, 555/288	17	0.193
ST4	195 C→T, 1693 C→T, 1799 C→T	3	SK-102, Vollum 1B, Vollum, CDC_684, 15(1345), 11(1940)	6	0.068
ST5	195 C→T, 1297 A→G, 1799 C→T	3	HYU01, I-364, 157(B-1107)	3	0.034
ST6	195 C→T	1	BA1035, SVA11	2	0.023
ST7	1803 T→C	1	Ohio ACB	1	0.011
ST8	195 C→T, 196 T→C, 1799C→T	3	2002013094	1	0.011
ST9	195 C→T, 1765 C→A	2	Kruger_B	1	0.011
ST10	195 C→T, 196 T→C, 869 T→G, 1799 C→T	4	*B. cereus* biovar anthracis CI, *B. cereus* G9241	2	0.023
ST11	17T→C, 59G→A, 95A→C, 195 C→T, 196T→C, 869T→G, 1799 C→T	7	*B. cereus* 03BB102	1	0.011

**Table 3:** Sequence types of the gene *lef*.

Sequence Type	Description of the Mutation	Number of Mutations in ST	List of Strains	Number of Strains	The Frequency of ST
ST1	-	0	14RA5914, A16, Stendal, Larissa, Shikan-NIID, A1144, Canadian bison, Ames Ancestor, BA1015, SK 102, Ohio ACB, Vollum1B, K3, Turkey32, Pollino, Vollum, A0248, CDC684, A2012, London 499, Kanchipuram, CZC5, 1199, 53169, LP51/4YA, I-271, 34(738), 15(1345), 1259, 1273, 52/33, 331/214, 822/7, 8(2099), 11(1940), 1173, A16R, V770-NP-1R, PAK 1, 644/268, 1055/38, 592/10, STI-1, K1285, A3716, A0135, A2075, A2079	48	0.545
ST2	2126A→G	1	1(14) Stavropol, LP50/3YA, 68/12, 367/17, 531/17, 7(992), 914/213, 1298, 1030/213, 1183, 219/6, 1056/51, 46/27, 546/714, 555/288, 47/28, 48/29	17	0.193
ST3	895G→A, 2126A→G	2	Tyrol4675, BA1035, RA3, HYU01, SVA11, 17OD930, BF1, Kruger_B, LP53/5YA, I-364, 44, 157(B-1107), Yamal 2	13	0.148
ST4	895G→T	1	Tangail-1, FDAARGOS 341, SPV842 15, Sterne, BFV	5	0.057
ST5	1036C→G	1	H9401	1	0.011
ST6	747A→C, 892C→A, 1628G→A, 2041G→A	4	2002013094	1	0.011
ST7	196G→A, 736G→A, 892C→A, 1046A→C, 1175G→A, 1216A→G, 1218A→G, 1628G→A, 1788G→A, 2041G→A	10	*B. cereus* biovar anthracis CI	1	0.011
ST8	892C→A, 1046A→C, 1216A→G, 1218A→G, 1628G→A, 2041G→A	6	*B. cereus* 03BB102	1	0.011
ST9	892C→A, 895G→, T 1046A→C, 1216A→G, 1218A→G, 1291T→G, 1292T→C, 1294A→G, 1305G→A, 1314T→C, 1316T→C, 1318G→A, 1336A→G, 1341G→T, 1385G→A, 1408T→G, 1628G→A, 1688A→C, 1689G→A, 1695A→G, 1840A→G, 1854C→G, 1897T→C, 1901T→C, 1904T→C, 1916G→T, 2030C→A, 2035C→G, 2041G→A, 2054A→T, 2064A→G, 2079T→C, 2084A→C, 2101C→T, 2104C→T, 2113T→C, 2128T→A, 2180A→C, 2385T→C	39	*B. cereus* G9241	1	0.011

**Table 4:** Sequence types of the gene *cya*.

Sequence Type	Description of the Mutation	Number of Mutations in ST	List of Strains	Number of Strains	The Frequency of ST
ST1	-	0	14RA5914, A16, Tangail-1, Stendal, Shikan-NIID, Ames Ancestor, BFV, A0248, A2012, I-271, 34(738), 52/33, SPV842 15, Sterne, A16R, FDAARGOS 341, A2079	17	0.193
ST2	600C→T	1	Larissa, A1144, BA1015, SK-102, Ohio ACB, Vollum 1B, Turkey32, Pollino, Vollum, CDC 684, London 499, 1(14)Stavropol, LP50/3YA, 68/12, LP51/4YA, 367/17, 531/17, 7(992), 1199, 53169, 1056/51, 15(1345), 1259, 1273, 46/27, 331/214, 546/714, 555/288, 822/7, 1030/213, 1183, 219/6, 8(2099), 11(1940), 47/28, 48/29, 1173, 914/213, 1298, V770-NP-1R, PAK-1, 644/268, 1055/38, 592/10, STI1, Kanchipuram, K1285, A3716, A0135	49	0.557
ST3	539A→G, 600C→T, 953T→C	3	Tyrol 4675, BA1035, RA3, HYU01, SVA11, 17OD930, BF1, Kruger_B, 44, 157(B-1107), I-364, LP53/5YA, Yamal 2	13	0.148
ST4	600C→T, 2129A→C	2	K3, CZC5, A2075	3	0.034
ST5	600C→T, 1140C→T	2	H9401	1	0.011
ST6	600C→T, 1329A→T, 1400A→G	3	Canadian bison	1	0.011
ST7	600C→T, 832A→G, 876G→A, 953T→C, 1971C→T, 2367T→A	6	2002013094	1	0.011
ST8	600C→T, 832A→G, 953T→C, 2081T→C, 2367T→A	5	*B. cereus* biovar anthracis CI, *B. cereus* G9241	2	0.023
ST9	600C→T, 832A→G, 953T→C, 2081T→C, 2264 G→T, 2367T→A	6	*B. cereus* 03BB102	1	0.011

**Table 5:** Predicted amino acid substitution in the PA protein.

*pagA* Mutation	Predicted Amino Acid Substitution in the Unprocessed PA	Domain of PA	Number of Strains
17T→C	6V→A	Signal peptide	1
59G→A	20S→N	Signal peptide	1
95A→C	32K→T	I	1
195C→T	- *		64
196T→C	66S→P	I, 1β2	4
869T→G	290I→S	II	3
981A→T	-		17
1297A→G	433I→V	II	3
1693C→T	565P→S	III	6
1765C→A	589Q→K	III	1
1799C→T	600A→V	III	61
1803T→C	-		1

* synonymous mutation.

**Table 6:** Predicted amino acid substitution in the LF protein.

*lef* Mutation	Predicted Amino Acid Substitution in the Unprocessed LF	Domain of LF	Number of Strains
196G→A	66E→K	I, 1α1	1
736G→A	246V→I	I, 1α9	1
747A→C	-		1
892C→A	298L→M	II, 2α1	4
895G→A	299A→T	II, 2α1	13
895G→T	299A→S	II, 2α1	6
1036C→G	346Q→E	III, 3α1 *, R2	1
1046A→C	349K→T	III, 3α1 *, R2	3
1175G→A	392R→K	III, 3α3, R4	1
1216A→G	406K→E	III, 3α4, R5	3
1218A→G	-		3
1291T→G	431L→A	II	1
1292T→C	-		1
1294A→G	432I→V	II	1
1305G→A	-		1
1314T→C	-		1
1316T→C	439L→P	II, 2α4	1
1318G→A	440D→N	II, 2α4	1
1336A→G	446K→E	II, 2α4	1
1341G→T	447R→S	II, 2α4	1
1385G→A	462S→N	II	1
1408T→G	470L→V	II, 2β1	1
1628G→A	543R→Q	II, 2β5	4
1688A→C	563K→T	II, 2β7	1
1689G→A	564K→T	II, 2β8	1
1695A→G	-		1
1788G→A	-		1
1840A→G	614K→E	IV	1
1854C→G	618F→L	IV, 4β1	1
1897T→C	633Y→H	IV, 4α2	1
1901T→C	634L→S	IV, 4α2	1
1904T→C	635I→T	IV, 4α2	1
1916G→T	639W→L	IV, 4α2	1
2030C→A	677T→K	IV, L2	1
2035C→G	679Q→E	IV, L2	1
2041G→A	681E→K	IV, L2	4
2054A→T	685Q→L	IV, L2	1
2064A→G	-		1
2079T→C	-		1
2084A→C	695E→A	IV	1
2101C→T	701L→F	IV, 4β4	1
2104C→T	702H→Y	IV, 4β4	1
2113T→C	705S→P	IV, 4β4	1
2126A→G	709E→G	IV	30
2128T→A	710L→I	IV	1
2180A→C	727D→A	IV, 4α4	1
2385T→C	-		1

* synonymous mutation.

**Table 7:** Predicted amino acid substitution in the EF protein.

*lef* Mutation	Predicted Amino Acid Substitution in the Unprocessed LF	Domain of EF	Number of Strains
539A→G	D→G180	PA-binding domain	13
600C→T	- *		71
832A→G	K→E278	PA- binding domain	4
876G→A	-		1
953T→C	I→T318	CA	17
1140C→T	-		1
1329A→T	E→D443	CB	1
1400A→G	E→G467	CB	1
1971C→T	-		1
2081T→C	V→A694	Spiral domain	3
2129A→C	H→P710	Spiral domain	3
2264G→T	R→M755	Spiral domain	1
2367T→A	N→K789	Spiral domain	4

* synonymous mutation.

**Table 8:** Separation of the studied strains into genotypes based on the *pagA*, *lef*, *cya*, and *atxA* ST combination.

Genotype	List of Strains	Number of Strains
GT1	Ames Ancestor, A0248, A16, Shikan-NIID, 14RA5914, A2012, 34(738), I-271, A16R, 52/33, Stendal	11
GT2	644/268, A0135, PAK-1, Turkey32, LP51/4YA, 53169, STI-1, BA1015, 1273, 8(2099), Larissa, Pollino, V770-NP-1R, 592/10, A1144, K1285, London 499, 1055/38	18
GT3	46/27, 1030/213, 914/213, 48/29, 1056/51, 1(14)Stavropol, 219/6, 1298, 531/17, 1183, 47/28, 555/288, 68/12, 546/714, 367/17, LP50/3YA, 7(992)	17
GT4	1259, 331/214, 822/7, 1199, 1173, Kanchipuram, A3716	7
GT5	LP53/5YA, Yamal 2, 44, 17OD930, Tyrol 4675, RA3, BF1	7
GT6	CDC_684, Vollum 1B, Vollum, 11(1940), 15(1345), SK-102	6
GT7	Sterne, SPV842_15, BFV, FDAARGOS 341, Tangail-1	5
GT8	A2075, K3, CZC5	3
GT9	HYU01, I-364, 157(B-1107)	3
GT10	BA1035, SVA11	2
GT11	A2079	1
GT12	H9401	1
GT13	Canadian bison	1
GT14	Ohio ACB	1
GT15	Kruger_B	1
GT16	2002013094	1
GT17	*B. cereus biovar anthracis* CI	1
GT18	*B. cereus* 03BB102	1
GT19	*B. cereus* G9241	1

**Table 9:** Presence of SNP *pagA* 1803T→C in strains of the A.Br.Aust94 group of American origin.

WGS Data Access Number	Strain	SNP *pagA* 1803T→C	Location
SRR1739963	2000031027	-	USA
SRR2339639	2000032893	-	USA
SRR2340252	2002734034	-	USA
SRR2340480	2002734153	-	USA
SRR5811018	2002734167	-	USA
SRR5811121	2002734054	-	USA
SRR5811124	2002734036	-	USA
SRR5811125	2002734037	-	USA
SRR2339643	2002013170	+	USA
SRR2340230	2002721571	+	USA

**Table 10:** Presence of SNP *lef* 895G→T in strains of the A.Br.001/002 group of different geographical origin.

WGS Data Access Number	Strain	SNP *lef* 895G→T	Location
CA_002007035	Brazilian Vaccinal	+	Brazil
SRR1739979	2000032979	+	USA
SRR5811142	2011218264	+	USA
ERR1596542	ANSES_08-07	+	France
ERR1596543	ANSES_08-09	+	France
ERR1596544	ANSES_08-10	+	France
ERR1596545	ANSES_08-11	+	France
ERR1596546	ANSES_08-13	+	France
ERR1596547	ANSES_08-14	+	France
ERR1596548	ANSES_08-15	+	France
ERR1596549	ANSES_08-16	+	France
ERR1596550	ANSES_08-19	+	France
ERR1596551	ANSES_08-20	+	France
ERR1596552	ANSES_08-21-1	+	France
ERR1596553	ANSES_08-21-2	+	France
ERR1596554	ANSES_08-21-3	+	France
ERR1596555	ANSES_08-22-2	+	France
ERR1596556	ANSES_08-24	+	France
ERR1596557	ANSES_08-25	+	France
ERR1596558	ANSES_08-26	+	France
ERR1596559	ANSES_08-27	+	France
ERR1596560	ANSES_08-28	+	France
ERR1596561	ANSES_08-29	+	France
ERR1596590	ANSES_11-04	+	France
ERR930304	Strain C (DK9)	+	Denmark
ERR930299	K929	+	Denmark
SRR5810961	2008725092	+	Bangladesh
SRR5811059	2008724724	+	Bangladesh
SRR5811137	2008724999	+	Bangladesh
SRR5811143	2008724997	+	Bangladesh
SRR5811158	2008724832	+	Bangladesh
SRR5811175	3000015248	+	Bangladesh
SRR5811176	3000015250	+	Bangladesh
SRR5811188	3000015251	+	Bangladesh
ERR1841046	ANSES_052 (CIP 53.169)	-	France
ERR1841047	ANSES_054 (CIP 81.89)	-	France
ERR1841049	ANSES_058 (CIP A211)	-	France
ERR930302	Strain A	-	Denmark
ERR930303	Strain B (DK8)	-	Denmark
SRR5811212	2002734373	-	Hong Kong
GCA_000219895	A0389 (ABLB)	-	Indonesia
SRR5811167	2000031042	-	Pakistan

## Data Availability

All data used for this study are available in the text of the article and in the Supplementary Materials. Additional data including generated genomic sequences of this study are available from the Russian Federal Service for Surveillance on Consumer Rights Protection and Human Wellbeing (Rospotrebnadzor) but restrictions apply to the availability of these data, which were used under license for the current study, and so are not publicly available. Data are however available from the authors upon reasonable request and with permission of the Russian Federal Service for Surveillance on Consumer Rights Protection and Human Wellbeing (Rospotrebnadzor).

## Supplementary Materials

The following are available online at https://www.mdpi.com/article/10.3390/pathogens10121556/s1, Table S1. Distribution of the studied strains by the MVLST-pXO1 genotypes (MVLST-pXO1-GT) depending on the combination of *pagA*, *lef*, *cya*, and *atxA* gene sequence types (ST).
