# Supplementary material for: Sequence Variability of pXO1-Located Pathogenicity Genes of Bacillus anthracis Natural Strains of Different Geographic Origin
Source: Pathogens. 2021 Nov 29;10(12):1556. doi: 10.3390/pathogens10121556 (PMC8703917; doi:10.3390/pathogens10121556)
Supplement: Supplementary file 1 [file pathogens-10-01556-s001.zip › pathogens-1448533-supplementary.pdf]

Table S1. Distribution of the studied strains by the MVLST-pXO1 genotype (MVLST-pXO1-GT) depending on the combination of pagA, lef, cya, and atxA gene sequencetypes (ST).

Strain*	Collection**	Location	canSNP group	*pagA*-ST	*lef*-ST	*cya*-ST	ST-atxA	MVLST-pXO1-GT
I-271	SRCAMB	Russia: Yamal Peninsula	A.Br.001/002	1	1	1	1	1
34(738)	SRCAMB	Kazakhstan	A.Br.001/002	1	1	1	1	1
52/33	SRCAMB	Russia: Chechen Republic	A.Br.001/002	1	1	1	1	1
14RA5914		Germany	A.Br.001/002	1	1	1	1	1
A16		China	A.Br.001/002	1	1	1	1	1
Stendal		Germany	A.Br.001/002	1	1	1	1	1
A16R		China	A.Br.001/002	1	1	1	1	1
Shikan-NIID		Japan	A.Br.Ames	1	1	1	1	1
Ames Ancestor		USA	A.Br.Ames	1	1	1	1	1
A0248		USA	A.Br.Ames	1	1	1	1	1
A2012		USA	A.Br.Ames	1	1	1	1	1
1273	SRCAMB	Russia: Volgograd region	A.Br.008/011	2	1	2	1	2
53169	SRCAMB	missing	A.Br.008/011	2	1	2	1	2
1055/38	SRCAMB	Russia: Samara region	A.Br.008/011	2	1	2	1	2
592/10	SRCAMB	Moldova	A.Br.008/011	2	1	2	1	2
644/268	SRCAMB	Ukraine	A.Br.008/011	2	1	2	1	2
8(2099)	SRCAMB	Russia: Tatarstan	A.Br.008/011	2	1	2	1	2
STI-1	SRCAMB	the laboratory strain	A.Br.008/011	2	1	2	1	2
LP51/4YA	SRCAMB	Russia: Yakutia	A.Br.008/011	2	1	2	1	2
BA1015		USA	A.Br.003/004	2	1	2	1	2
V770-NP-1R		USA	A.Br.003/004	2	1	2	1	2
A0135		Tanzania	A.Br.005/006	2	1	2	1	2
Larissa		Greece	A.Br.008/011	2	1	2	1	2
Turkey32		Turkey	A.Br.008/011	2	1	2	1	2
PAK-1		Pakistan	A.Br.008/011	2	1	2	1	2
K1285		Namibia	A.Br.008/011	2	1	2	1	2
A1144		Argentina	A.Br.011/009	2	1	2	1	2
Pollino		Italy	A.Br.011/009	2	1	2	1	2
London 499		Great Britain	A.Br.011/009	2	1	2	1	2
1(14) Stavropol	SRCAMB	Ukraine	A.Br.008/011	3	2	2	1	3

1030/213	SRCAMB	Russia: Karachay-Cherkessia	A.Br.008/011	3	2	2	1	3
1056/51	SRCAMB	Russia: Stavropol territory	A.Br.008/011	3	2	2	1	3
219/6	SRCAMB	Uzbekistan	A.Br.008/011	3	2	2	1	3
367/17	SRCAMB	Russia: Tula region	A.Br.008/011	3	2	2	1	3
46/27	SRCAMB	Russia: Chechen Republic	A.Br.008/011	3	2	2	1	3
47/28	SRCAMB	Russia: Chechen Republic	A.Br.008/011	3	2	2	1	3
48/29	SRCAMB	Russia: Chechen Republic	A.Br.008/011	3	2	2	1	3
531/17	SRCAMB	Russia: Kalmyk Re-public	A.Br.008/011	3	2	2	1	3
546/714	SRCAMB	Russia: Voronezh region	A.Br.008/011	3	2	2	1	3
555/288	SRCAMB	Russia: Orenburg region	A.Br.008/011	3	2	2	1	3
68/12	SRCAMB	Azerbaijan	A.Br.008/011	3	2	2	1	3
7(992)	SRCAMB	Russia: Novgorod region	A.Br.008/011	3	2	2	1	3
914/213	SRCAMB	Russia: Chechen Republic	A.Br.008/011	3	2	2	1	3
LP50/3YA	SRCAMB	Russia: Yakutia	A.Br.008/011	3	2	2	1	3
1183	SRCAMB	Russia: Kabardino-Balkar Republic	A.Br.008/011	3	2	2	1	3
1298	SRCAMB	Russia: Volgograd region	A.Br.008/011	3	2	2	1	3
1173	SRCAMB	Russia: Stavropol territory	A.Br.Aust94	1	1	2	1	4
1259	SRCAMB	Russia: Stavropol territory	A.Br.Aust94	1	1	2	1	4
1199	SRCAMB	Russia: Dagestan	A.Br.Aust94	1	1	2	1	4
331/214	SRCAMB	Azerbaijan	A.Br.Aust94	1	1	2	1	4
822/7	SRCAMB	Russia: Chechen Republic	A.Br.Aust94	1	1	2	1	4
Kanchipuram		India	A.Br.Aust94	1	1	2	1	4
A3716		Namibia	A.Br.Aust94	1	1	2	1	4
LP53/5YA	SRCAMB	Russia: Yakutia	B.Br.001/002	2	3	3	1	5

Yamal-2	SRCAMB	Russia: Yamal Pen-insula	B.Br.001/002	2	3	3	1	5
44	SRCAMB	missing	B.Br.CNEVA	2	3	3	1	5
Tyrol 4675		Austria	B.Br.CNEVA	2	3	3	1	5
RA3		France	B.Br.CNEVA	2	3	3	1	5
BF1		Germany	B.Br.CNEVA	2	3	3	1	5
17OD930		Switzerland	B.Br.CNEVA	2	3	3	1	5
11(1940)	SRCAMB	Turkmenistan	A.Br.Vollum	4	1	2	1	6
15(1345)	SRCAMB	Tajikistan	A.Br.Vollum	4	1	2	1	6
SK-102		USA	A.Br.Vollum	4	1	2	1	6
Vollum 1B		USA	A.Br.Vollum	4	1	2	1	6
Vollum		USA	A.Br.Vollum	4	1	2	1	6
CDC 684		USA	A.Br.Vollum	4	1	2	1	6
Tangail-1		Bangladesh	A.Br.001/002	1	4	1	1	7
BFV		Jamaica	A.Br.001/002	1	4	1	1	7
FDAARGOS 341		USA	A.Br.001/002	1	4	1	1	7
SPV842 15		Brazil	A.Br.001/002	1	4	1	1	7
Sterne		USA	A.Br.001/002	1	4	1	1	7
K3		South Africa	A.Br.005/006	2	1	4	1	8
CZC5		Zambia	A.Br.005/006	2	1	4	1	8
A2075		Tanzania	A.Br.005/006	2	1	4	1	8
157(B-1107)	SRCAMB	Estonia	B.Br.001/002	5	3	3	1	9
I-364	SRCAMB	Russia: Buryatia	B.Br.001/002	5	3	3	1	9
HYU01		Korea	B.Br.001/002	5	3	3	1	9
BA1035		South Africa	B.Br.001/002	6	3	3	1	10
SVA11		Sweden	B.Br.001/002	6	3	3	1	10
A2079		Tanzania	A.Br.005/006	2	1	1	1	11
H9401		Korea	A.Br.005/007	2	5	5	1	12
Canadian bison		Canada	A.Br.WNA	2	1	6	1	13
Ohio ACB		USA	A.Br.Aust94	7	1	2	1	14
Kruger B		South Africa	B.Br.Kruger	9	3	3	1	15
2002013094		USA	C.Br.001	8	6	7	1	16
Bacillus cereus biovar anthracis CI		Ivory Coast	-	10	8	8	2	17
Bacillus cereus 03BB102		USA	-	11	7	9	1	18
Bacillus cereus G9241		USA	-	10	9	8	1	19

* strains belong to *B. anthracis*, unless otherwise specified

** The presence of the strain in the SRCAMB collection is indicated.

* strains belong to B. anthracis, unless otherwise specified.

** The presence of the strain in the SRCAMB collection is indicated.
